# Could Public Restrooms Be an Environment for Bacterial Resistomes?

**DOI:** 10.1371/journal.pone.0054223

**Published:** 2013-01-17

**Authors:** Hermine V. Mkrtchyan, Charlotte A. Russell, Nan Wang, Ronald R. Cutler

**Affiliations:** 1 Pre-Clinical Drug Discovery Group, School of Biological and Chemical Sciences, Queen Mary University of London, London, United Kingdom; 2 School of Biological and Chemical Sciences, Queen Mary University of London, London, United Kingdom; 3 School of Life Science and Technology, University of Electronic Science and Technology, Chengdu, China; University of Iowa Carver College of Medicine, United States of America

## Abstract

Antibiotic resistance in bacteria remains a major problem and environments that help to maintain such resistance, represent a significant problem to infection control in the community. Restrooms have always been regarded as potential sources of infectious diseases and we suggest they have the potential to sustain bacterial “resistomes”. Recent studies have demonstrated the wide range of different bacterial phyla that can be found in non-healthcare restrooms. In our study we focused on the Staphylococci. These species are often skin contaminants on man and have been reported as common restroom isolates in recent molecular studies. We collected samples from 18 toilets sited in 4 different public buildings. Using MALDI-TOF-MS and other techniques, we identified a wide range of antibiotic resistant Staphylococci and other bacteria from our samples. We identified 19 different Staphylococcal species within our isolates and 37.8% of the isolates were drug resistant. We also identified different Staphylococcal species with the same antibiograms inhabiting the same restrooms. Bacterial “resistomes” are communities of bacteria often localised in specific areas and within these environments drug resistance determinants may be freely transferred. Our study shows that non-healthcare restrooms are a source of antibiotic resistant bacteria where a collection of antibiotic resistance genes in pathogenic and non-pathogenic bacteria could form a resistome containing a “nexus of genetic diversity”

## Introduction

Molecular techniques have recently been used to demonstrate the wide range of bacterial phyla that can be found in public restrooms [1]. Previous investigations of restrooms in non-healthcare environments concentrated primarily on investigating contamination with bacteria from faecal and/or skin origin [2]-[5] and in hospitals the focus was on *Staphylococcus aureus* or MRSA [6]. The recent study by Flores and co-workers [1] however, demonstrated the wide diversity of bacterial phyla that can be present in public restrooms and indicated that these phyla were usually related to bacteria associated with man. It is therefore not surprising that organisms associated with the human “microbiome” [7] should have an impact on the microbial flora in restrooms [1].

Apart from the presence of human pathogens in restroom environments, there is also the possibility that restroom environments could harbor antibiotic resistant bacteria, as other studies looking at non-hospital environments with equally diverse bacterial populations, have suggested that such populations can provide effective environments to aid the development, sustainability and spread of antibiotic resistance in bacteria [8]. In addition to this there is also the suggestion that cells can survive or persist in such environments even if there are restrictions on resources [9].

It has been proposed that human society’s overuse and abuse of antibiotics is the main factor in the development and sustainability of antibiotic resistance in bacteria and that to restore the efficacy of antibiotics, this ecological balance had to be adjusted to favor antibiotic susceptible bacteria rather than antibiotic resistant bacteria [10], [11]. However, despite the huge efforts that have been made to control and reduce antibiotic use and misuse in man and in animals [6], [12], antibiotic resistance in bacteria continues to spread and cause morbidity, mortality and increasing costs in the treatment of infectious diseases [12], [13].

In addition to problems with antibiotic misuse however, another factor which could also be involved with the persistence of drug resistance in bacteria in the environment, is their ability to form “resistomes”, closely associated groups of bacteria able to share and maintain drug-resistance determinants within suitable environments [14]–[16]. Bacterial resistomes may be associated with interactions between a wide range of different bacterial species or even interactions locally between organisms of the same species [11], [16], [17].

In our study, the *Staphylococcaceae*, which are commonly found associated with restrooms [1], were selected for further study. However, to fully evaluate the propensity of antimicrobial resistance in this group of organisms, identification to species level is required. In the past, full identification of environmental bacterial species could be difficult and time-consuming as the majority of high-throughput bacteriological identification systems were developed for identifying hospital isolates. In our study we additionally evaluated the use of MALDI-TOF-MS [18], [19] for identifying environmental isolates and compared this method to selected routine and molecular methods.

## Materials and Methods

### Sample Collection

Dry sterile cotton swabs (Copan Diagnostics Inc., USA) were used to collect samples from 18 randomly selected public restrooms (non-healthcare) in London United Kingdom. Sampling was carried out in different buildings and over a period of 24 weeks. 21 sites were sampled in each restroom. All specimens were transferred to the laboratory within 1-3 hrs.of the sample being taken. In the laboratory, swabs were suspended in 1ml sterile 0.9% saline, inoculated directly onto Nutrient Agar (Nutrient Agar, Oxoid, Basingstoke, UK) and plates were incubated aerobically at 37°C for 24-48 h.

### Identification of the Environmental Isolates

#### Conventional and biochemical methods

These methods were used for Gram-positive cocci and Gram-negative rods only. Gram-positive cocci were provisionally identified by conventional methods including catalase, coagulase tests and selective media, (Mannitol Salt Agar, Oxoid Ltd, Basingstoke, UK), Gram negative rods were provisionally identified using selective media (BRILLIANCE™ UTI selective agar, Oxoid Ltd., Basingstoke, UK).

Gram positive cocci were additionally characterised to species level using the API ID 32 STAPH system (BioMerieux Ltd., Marcy l’Etoil, France) according manufacturer’s instructions and the Prolex™ Staph Xtra Latex Kit was used to distinguish *Staphylococcus aureus* and methicillin-resistant *S. aureus* (MRSA) from other species of *staphylococci* (Prolab Diagnostics, Neston, South Wirral, UK).

#### Partial 16S RNA gene sequencing for Staphylococci

Genomic DNA of the isolates was prepared using a commercial kit (Qiagen, Crawley, UK). Staphylococci were subjected to partial 16S rRNA gene sequencing using primers described previously [20]. PCR thermal cycling conditions were 5 min at 94°C, 30 cycles for 30 sec at 94°C, 1 min for 50°C and 30 sec for 72°C. The 2 log DNA ladder I (New England Biolab, Hitchin, UK) was used as molecular size markers. Amplified PCR products were sequenced by Eurofins MWG GmBH (Ebersberg, Germany) using ABI 3730×L DNA analyser.

#### MALDI-TOF-MS analysis

All isolates were purified and analysed using Matrix-assisted laser desorption ionization time-flight mass-spectroscopy (Microflex LT, MALDI-TOF-MS, Bruker Daltonics, Coventry, UK) in a positive linear mode (2000 to 20000 m/z range). The resulting spectra for each culture was analysed by MALDI-Biotyper 2.0 software (Bruker Daltonics, Coventry, UK). The software evaluates each spectra compared to a reference spectra in the Bruker Taxonomy Database identifying the best match from database records. Results were expressed as scores (QI) from 0 to 3, as recommended by the manufacturer. Scores QI ≤1.7 were not considered as reliable identification. A score of QI≥1.7 corresponded to ‘genus’ identification. Only scores higher than QI≥2 were considered a reliable identification of species. MALDI-TOF-MS identifications were performed in duplicate using extracted and direct methods as recommended by the manufacturer. *E. coli* DH5 (Bruker Daltonics, Coventry, UK) was used as a standard for calibration and quality control.

#### MALDI-TOF-MS analysis – Direct method

A single colony of each overnight culture was transferred onto a MALDI-TOF-MS ground steel target plate using a disposable loop and dried for five minutes at room temperature. The HCCA matrix solution (1 µl) was overlaid onto each target spot.

#### Extracted method

3-5 colonies of overnight cultures were suspended in 300 µl distilled water. The suspension was mixed with 900 µl absolute ethanol and centrifuged for 2min at 13000×g. The pellets were re-suspended in 25 µl of 70% formic acid and then 25 µl pure acetonitrile was added. After mixing solutions were centrifuged at 13000×g for 2 min. 1 µl aliquots of the supernatant were spotted in duplicate onto MALDI ground steel targets, dried in air for 5 min at room temperature and each target spot was overlaid with 1 µl α-cyano-4-hydroxycinnamic (HCCA) matrix solution.

### Antimicrobial Susceptability Testing

Three culture based methods were used to screen for antibiotic susceptibilities of Gram-positive cocci and Gram-negative rods. Mastrings and Microscan Walkaway Plus. Zones of inhibition were evaluated using Mastring M13 were used for Gram positives cocci and Mastring M14 for Gram negative rods according to manufacturers instructions (Mast Diagnostics, Merseyside, UK). For Gram-positives the minimum inhibitory concentrations (MIC) were determined using the MicroScan Walkway 96 plus automated system (Siemens Healthcare Diagnostics, CA, USA). The MICs to oxacillin were additionally evaluated using “M.I.C. evaluators”, antimicrobial gradient strips designed for accurate Minimum Inhibitory Concentration (MIC) values (Oxoid Ltd., Basingstoke, UK).

For Gram-negative bacteria the minimum inhibitory concentrations (MIC) were also determined using the MicroScan Walkway 96 plus automated system (Siemens Healthcare Diagnostics, CA, USA).

### Detection of PBP2'

For resistant staphylococci a rapid latex agglutination assay kit, the Penicillin-binding protein (PBP2’) latex agglutination test was used (according the manufacturer’s instructions) to determine PBP2' (Oxoid Ltd., Basingtoke, UK).

### PCR Amplification

Genomic DNA of the isolates were prepared using commercial kits, QIA amp DNA mini kit (Qiagen, Crawley, UK). *SCCmec* type was determined by detecting *mec* and *ccr* complexes using the primers as described previously [21]. PCR thermal cycling conditions were 5 min at 94°C, 30 cycles for 30 sec at 94°C, 1 min for 50°C and 30 sec for 72°C. The 2 log DNA ladder I (New England Biolab, Hitchin, UK) was used as molecular size markers.

## Results

### Samples

Of the 21 sites sampled in each restroom we identified 6 sites which were the most contaminated. These were the hand dryer systems, toilet seats, inner door surfaces, taps, soap dispensers and urinal floors.

### MALDI-TOF-MS Analysis

211 of the 256 environmental isolates (82.4%) were identified using MALDI-TOF-MS (Table 1) however 17.6% of isolates failed to give a reliable identification. The rates of MALDI-TOF identification at the species level with a score of QI≥2 were 70.7% (149/211) and at genus level with a score QI≥1.7 but QI≤2.0 were 29.3% (62/211). Analysis using the direct method resulted in 163 isolates identified, 67.7% of these at species level and 37.3% at genus level respectively. The alcoholic extraction method significantly improved identification with 185 identified, 83.7% of these at species level and 16.3% at genus level respectively. This confirms that best practice for environmental isolates, as recommended by the manufacturer for clinical isolates, is to use both direct and extraction methods in combination.

**Table 1:** Summary of Family and Genera of bacteria identified by MALDI-TOF-MS.

Family		Genus	No of isolates
**Staphylococcaceae**		**Staphylococcus**	**103**
**Bacillaceae**		**Bacillus**	**37**
**Micrococcaceae**		**Micrococcus**	**30**
**Enterobacteriaceae**		**TOTAL** (composed of −)	**9**
*"*		*Escherichia*	*1*
*"*		*Proteus*	*5*
*"*		*Citrobacter*	*2*
*"*		*Morganella*	*1*
**Moraxellaceae**		**Acinetobacter**	**7**
**Corynebacteriaceae**		**Corynebacterium**	**4**
**Comamonadaceae**		**Delftia**	**2**
**Sphingobacteriaceae**		**Sphingobacteria**	**2**
**Campylobacteraceae**		**Campylobacter**	**1**
**Pseudomonacae**		**Pseudomonas**	**1**
**Others** *			**15**
**TOTAL**			**211**

* Others include genera of *Korucia (6); Rothia (2); Arthrobacter (2); Anaerococcus (3); Rhodococcus (2)*.

For the Gram negatives tested there was agreement between conventional methods, 16S DNA and MALDI-TOF-MS analysis for *E. coli* and other Gram-negative bacteria such as *Proteus mirabilis* and *Acinetobacter spp*. Both extracted and direct methods were effective in identifying *E. coli* and produced high scores of QI = 2.473 and QI = 2.341 respectively. The alcoholic extraction method increased identification of *Proteus* (80%) and *Acinetobacter* (85.7%) (Table 2).

**Table 2:** Concordance between conventional and direct and extracted MALDI-TOF-MS methods for Gram-negative isolates of three genera.

Genera	Total	Conventional	D-MALDI-TOF	E-MALDI-TOF	AE increase(%)
*E.coli*	1	1	1	1	n/a
Proteus	5	5	1	4	80
Acinetobacter	7	7	1	6	85.7

D-MALDI-TOF (direct method); E-MALDI-TOF (extracted method); AE increase (increase of identification after alcoholic extraction).

In this current study we had a large number of staphylococci in our samples. We paid particular attention to evaluating the efficiency of MALDI-TOF to identify environmental staphylococci and compared the results to conventional identification tests, (API ID 32 STAPH tests and PCR sequencing). Using the AE method with MALDI-TOF-MS increased the identification rate for staphylococci at species level and produced the highest MALDI-TOF-MS QI score, 2.437. Overall, we identified 103 staphylococcal isolates belonging to 15 species. This included *S. aureus, Staphylococcus hominis, Staphylococcus haemolyticus, Staphylococcus warneri, Staphylococcus pasteuri, Staphylococcus simulans, Staphylococcus saprophyticus, Staphylococcus epidermidis, Staphylococcus cohnii, Staphylococcus capitis, Staphylococcus pettenkoferi, Staphylococcus lugdunensis, Staphylococcus arlettae, Staphylococcus equorum, Staphylococcus sciuri*. When we compared the MALDI-TOF-MS and PCR sequencing identifications to those obtained by the API ID 32 STAPH system, the API ID 32 STAPH system misidentified *S. pasteuri* isolates as *S. warneri* (one *S. pasteuri* was identified as *S. hominis*) and *S. warneri* isolates as *S. saprophyticus.* One *S. haemolyticus* was identified as *S.hominis* and one *S. epidermidis* was identified as *S. capitis.* Only one staphylococcal isolate (*S. cohnii*) was misidentified by MALDI-TOF-MS while PCR sequencing results identified as it *S. haemolyticus* and by API test as *S. xylosus* (Table 3).

**Table 3:** Identification of Staphylococci isolates by MALDI-TOF MS and API ID 32 STAPH system compared with 16S RNA sequencing.

MALDI-TOF MS	API ID 32 STAPH	PCR
*S. pasteuri*	*S. warneri*	*S. pasteuri*
*S. pasteuri*	*S. hominis*	*S. pasteuri*
*S. warneri*	*S. saprophyticus*	*S. warneri*
*S. haemolyticus*	*S. hominis*	*S. haemolyticus*
*S. epidermidis*	*S. capitis*	*S. epidermidis*
*S. cohnii*	*S. xylosus*	*S. haemolyticus*

### Antibiotic Susceptibility

Antibiotic resistance was detected both in Gram positive and Gram negative isolates. Four out of 20 Gram-negative bacteria species identified were resistant to antibiotics, this included ampicillin, cephalothin, streptomycin, sulphatriad, tetracycline, cotrimoxazole (Table 4). In contrast, 39 staphylococcal isolates (37.8%) were drug resistant, including resistance to non-β-lactam antibiotics such as fusidic acid, gentamycin, erythromycin and chloramphenol (Table 5). In addition to these multiply antibiotic resistant strains, 5 of the staphylococcal strains isolated were resistant only to oxacillin and penicillin although their oxacillin MICs were amongst the highest identified, 64 mg/l or greater. The majority of other strains, although also *mecA* positive, had MICs of 4 mg/l or below. Overall MICs for oxacillin varied from 0.25 to 128 mg/l (Table 5).

**Table 4:** Profiles of resistant Gram-negative isolates found in restrooms.

Gram-negative isolates	wr/B		Resistance Profiles^ β^
*Sphingobacterium mizutaii*	1 K	Am, Co, KF, S, ST, TS	
*D.acidovorans*	11 K	A, Co, G, GM, N,S, ST, T, Tb	
*P.mirabilis*	12 K	Co, KF, ST, TS	
*E. coli*	18 K	Am, Am/S, GM, KF, S, ST, T,TS	
*A.baumannii*	18 K	Am, C, Ct, Cf, KF, S, ST, T,TS	

wr/B* restroom/building code G,K,H or T.

β - A: Amikacin; Am: Ampicillin; Am/S: Ampicillin/Sulbactam; KF: Cephalotoxin; S: Streptomycin; ST: Sulphatriad; T: Tetracycline; TS: Cotrimoxazole; Co: Collstin Sulphate C: Cefepime; Ct: Cefotaxime; Cf: Ceftazidime; G: Gentamicin; Tb: tobramycin; N: Netilmicin.

**Table 5:** Resistance profiles and molecular characterisation of antibiotic resistant Staphylococci isolated from 15 out of 18 different restrooms from 4 buildings (G,H,K,T).

Species	Wr/B	n	A	Am	Az	C	Cl	Cp	Cx	E	F	Fa	G	I	L	Mc	Mp	Mx	N	P	S	St	T	Tb	O MIC	MecA
*S. aureus*	1 K	1	R	R	R	R	R	R	R	R			R	R	R		R	R		R				R	2	+
*S. pasteuri*	2T	1		R						R		R								R	R	R	R		0.25	+
*S.epidermidis*	4T	1																		R					64	+
*S.haemolyticus*	4T	1		R	R	R	R			R	R		R	R			R			R					1	+
*S. pasteuri*	4T	1		R						R	R	R	R	R						R	R	R	R		0.75	+
*S. hominis*	4T	1	R	R	R	R	R		R	R	R	R					R			R					1	+
*S.haemolyticus*	5T	1		R	R	R	R			R	R		R	R			R			R					1	+
*S. hominis*	6 K	1		R						R		R	R							R	R		R		1.5	+
*S. aureus*	6 K	1	R	R	R	R	R	R	R	R		R	R			R	R	R		R				R	2	+
*S. simulans*	7G	1																		R					0.5	+
*S.saprophyticus*	7G	1		R								R	R						R	R	R		R		2	+
*S. warneri*	7G	2	R	R	R	R	R		R	R	F	R	R				R			R					1	+
*S. hominis*	9H	3		R						R		R	R							R	R		R		0.75–1.5	+
*S. equorum*	11 K	1																		R					64	+
*S. haemolyticus*	11 K	1								R		R								R	R		R		0.75	+
*S. warneri*	11 K	1		R						R		R								R	R		R		0.75	+
*S. hominis*	11 K	1		R						R		R	R							R	R		R		1	+
*S. warneri*	12 K	2		R						R										R	R				0.75	+
*S warneri*	13H	1																		R					128	+
*S.saprophyticus*	13H	1		R						R		R							R	R	R		R		1.5	+
*S. pasteuri*	14T	2		R						R		R	R							R	R		R		0.5	+
*S. pasteuri*	15 K	1								R		R								R					1	+
*S.saprophyticus*	15 K	1		R						R		R	R						R	R	R		R		4	+
*S. cohnii*	16 K	1		R						R		R								R	R		R		2	+
*S. aureus*	16 K	1		R						R		R	R							R	R		R		4	+
*S. epidermidis*	16 K	2		R						R		R	R							R	R		R		0.5–1.5	+
*S. haemolyticus*	16 K	3		R						R		R	R							R	R		R		0.5–1.5	+
*S. aureus*	17 K	1		R						R		R	R							R	R		R		64	+
*S. epidermidis*	18 K	1																		R					128	+
*S. capitis*	18 K	1								R		R								R	R				1	+
*S.saprophyticus*	18 K	1		R						R		R	R						R	R	R		R		4	+

n* similar isolates from each restroom but different sites.

wr/B* restroom/Building code G,K,H or T.

A: Amoxacillin; Am: Ampicillin; Az: Azitromycin; C: Cefepime; Cx: Cefuroxime:; Cp: ciprofloxacin; Cl: Clindamycin; E: Erythromycin; F: Fosfomycin; Fa: Fusidic Acid; G: Gentamicin; I: Imipenem; L: levofloxacin; Mp: Meropenem; Mx: Moxifloxacin; Mc: Mupiricin; N: Novobiocin; O: oxacillin; P: Penicillin; S: Streptomycin; T: Tetracycline. Tb: tobramycin.

In some cases there were up to 4 different staphylococcal species with closely related antibiograms isolated from different sites in the same restroom. This could be in keeping with the local spread of drug resistance determinants between related organisms present in the same restroom. In restroom 16 K for example, 7 antibiotic resistant staphylococcal strains were isolated from different sites in this restroom, these were of 4 different species but each with the same antibiogram. In addition two of these species had multiple isolates with the same antibiograms, *S.epidermidis* (2 isolates) and *S.haemolyticus* (3 isolates). Overall in restroom 16 K (table 5) the majority of strains carried 7 of the same class of antibiotic resistance determinants, (including mec A) common in all 5 isolates.

### PBP2′ Detection and PCR Amplification

In spite of the low MICs to methicillin in many staphylococcal species, (mentioned above) we identified *mecA* in all of isolates tested (Table 4).

## Discussion

Our knowledge as too the variety of bacterial species which can exist in microbiomes [7] is still developing and our understanding about the spread and dissemination of antibiotic resistance within such environments remains a challenge [8].

Bacteria shed from human skin, (*Propionibacteriaceae, Corynebacteriaceae, Staphylococcaceae and Streptococcaceae*) are common in restroom environments [1] and these are not only the coliforms normally targeted in hygiene screens [2], [4], [5]. In our study we isolated a wide range of bacterial species from public restrooms and found *Staphylococcaceae* were common in our study (Table 1) as they were in the study by Flores et al [1]. Because of this we selected non-healthcare restroom isolates of staphylococci to evaluate for their propensity of drug resistance.

Staphylococci were isolated from 18 restrooms often on different days. Previous studies have investigated non-healthcare restrooms and homes but these targeted enteric pathogens and did not evaluate levels of antibiotic resistance [2], [4], [5].

We propose that restrooms, especially with their continual influx of bacterial flora from man, could be non-healthcare environments for the collection of bacterial resistomes, which as defined by Wright are a “collection of antibiotic resistance genes in pathogenic and non-pathogenic bacteria”. Over a third of staphylococci isolated in our study carried antibiotic resistance determinants (Table 5).

Recently the bacterial phyla present in 12 selected restrooms were comprehensively determined in a study by Flores and co-workers [1]. They demonstrated that organisms potentially shed from human skin were most common in these environments in particular, *Propionibacteriaceae, Corynebacteriaceae, Staphylococcaceae* and *Streptococcaceae* and not the coliforms normally targeted in restroom hygiene screens [2], [4], [5].

It was important to identify staphylococci to species level for our study. For this we evaluated a number of methods. MALDI-TOF-MS produced an identification rate similar to that found with previous hospital studies (around 82 to 99%) [18], [20], [22]. PCR agreed with MALDI-TOF-MS (99%). However the API ID 32 STAPH only produced only 54% agreement with the other methods (Table 3). These figures were lower than those found using API systems in clinical studies, but many of the species we identified had not been evaluated in clinical studies [23]. Other studies using environmental or animal coagulase negative staphylococci (CoNS) have also reported poor identification using API systems [24], [25] [26].

Staphylococci are a major cause of nosocomial and community acquired infections and they can have a high intrinsic resistance to antimicrobials [27]. This potential problem could be augmented by fact that species traditionally regarded as hospital pathogens are also isolated from non-hospital environments. CoNS in particular, belong to this group [28]. We sampled 18 restrooms in 4 different buildings over a period of 24 weeks and found a wide variety of staphylococcal species, many of which were antibiotic resistant. The antibiograms of some of these species were closely associated with the antibiograms found in different species from the same restrooms on different dates and others with isolates from restrooms in the same building (table 5). We particularly found the widespread dissemination of drug resistance in CoNS from these restroom environments. The numbers of antibiotic resistance determinants carried by these strains varied from 1 to 15. The most common resistances were: Penicillin, found in 100% of isolates, Erythromycin, found in 90%, Amoxillin, 80% and Fusidic acid, 74%. These resistances were common to isolates in all four buildings sampled over the four weeks of the study.

Regarding the possibility of the direct transfer of resistance determinants within restrooms and/or within buildings, there were 11 staphylococcal isolates with the same antibiograms representing 5 different staphylococcal species and these were isolated from 5 different restrooms within the same building (Table 5). The species (and number of isolates) involved were *S.saprophyticus* (n = 3), *S.epidermidis* (n = 2), *S.hominis* (n = 2), *S.haemolyticus* (n = 3) and *S. aureus* (n = 1). The novobiocin resistance in *S.saprophyticus* strains was not included in the evaluation as this species is inherently resistant to this drug and no other isolates of Staphyloccocal species were found to be resistant [29].

A possible example of the transfer of drug resistance determinants occurring between different staphylococcal species in the same restroom is shown with 7 isolates in restroom 16 K. All 7 isolates were antibiotic resistant at some level, but 6 of the isolates, *S. haemolyticus* (n = 3), *S. epidermidis* (n = 2) and one *S. aureus,* which were isolated from different sites within the same restroom, had the same antibiogram. The two coagulase negative staphylococci, *S. haemolyticus* and *S. haemolyticus* also happen to be the two of the most common CoNS found on man [30]. It is possible that the 3 species with the same antibiogram came from the same human source, as individuals undergoing long-term treatment for acne can carry the more than one drug resistant staphylococcal species [31]. These strains could then have been transferred to different sites within the same restroom through poor hygiene practices. However, this particular antibiogram was also found in other Staphylococcal species isolated from 4 other restrooms within the same building on different days, potentially indicating a widespread dissemination of these resistance determinants through different staphylococcal species and restrooms.

Other examples of similarities of antibiotic resistance determinants within these environments were found. Isolates of *S.saprophyticus,* for example were found with the same antibiograms in different restrooms in the same building. Three *S.hominis* strains were isolated from the same restroom and they also had the same antibiogram and 2 *S. pasteuri* with the same antibiogram were also isolated from different sites from different restrooms in the same building. Although it is possible that contaminated individuals were spreading these drug resistance determinants throughout buildings, there is also the concern that antibiotic resistance is so common in these environments and that it is easily spread. It should also be noted that Coagulase-negative staphylococci can be a significant problem in healthcare situations and in some countries, have been reported to be the third most common causative agent of nosocomial infections and the most frequent cause of nosocomial bloodstream infections [32]-[34].

Our results showed that more than one third (37%) of staphylococcal species isolated from our restroom samples were carrying the *mecA* gene, although some had low MIC values to oxacillin. Low MICs to oxacillin have also been reported in clinical isolates of *S.aureus* [35] and *S. haemolyticus* [28] and these isolates have also been reported as *mec*A positive.

Our findings, in common with those mentioned above, demonstrate a commonality between low levels of oxacillin resistance in clinical and/or in environmental isolates and the carriage of *mec*A. By contrast, the five of the CoNS isolates, although not multidrug resistant still carried *mecA* and demonstrated high MIC values (64 and 128mg/l) to oxacillin. In this group with high oxacillin MICs were three strains of *S.epidermidis*, one *S.warneri* and one *S.equorum*. These strains were only resistant to oxacillin and penicillin. By comparison the only *S.aureus* strain in this “high resistance” group was multiply drug resistant. This level of oxacillin resistance has been also reported in community acquired MRSA [35]. CAMRSA is well established throughout the world, carriage and infections are often associated with close communities such as students, military personnel and athletes [36].

Kümmerer in 2004 [14] suggested that for the transfer of resistance to occur, bacteria should be able to survive in the environment and carry stable genetic material. There is also the proposal that antibiotic resistant bacteria can survive and persist even in harsh environments [9]. Our study shows that bacteria, not commonly associated with healthcare settings, carry resistance determinants. Non-healthcare restrooms are a source of antibiotic resistant bacteria. This shows the potential for public restroom “resistomes” to exist, where a collection of antibiotic resistance genes in pathogenic and non-pathogenic bacteria produce, as suggested by Wright, a “nexus of genetic diversity” [37].
